# Short-term Heart Rate Turbulence Analysis Versus Variability and Baroreceptor Sensitivity in Patients With Dilated Cardiomyopathy^1^

**Published:** 2004-10-01

**Authors:** Hagen Malberg, Robert Bauernschmitt, Udo Meyerfeldt, Alexander Schirdewan, Niels Wessel

**Affiliations:** * Forschungszentrum Karlsruhe GmbH (Karlsruhe Research Center), Institute for Applied Computer Science, P.O. Box 3640, D-76021 Karlsruhe, Germany. E-mail: malberg@iai.fzk.de; † German Heart Centre Munich, Department of Cardio-Vascular Surgery, Munich, Germany; ‡ Medical Faculty of the Charité, Franz Volhard Clinic, Helios Klinikum-Berlin, Germany; § University of Potsdam, Institute of Physics, Am Neuen Palais 10, D-14415 Potsdam, Germany

**Keywords:** Spontaneous Baroreflex, Heart Rate Turbulence, Heart Rate Variability, Blood Pressure Variability, Dilated Cardiomyopathy (DCM)

## Abstract

New methods for the analysis of arrhythmias and their hemodynamic consequences have been applied in risk stratification, in particular to patients after myocardial infarction. This study investigates the suitability of short-term heart rate turbulence (HRT) analysis in comparison to heart rate and blood pressure variability as well as baroreceptor sensitivity analyses to characterise the regulatory differences between patients with dilated cardiomyopathy (DCM) and healthy controls. In this study, 30 minutes data of non-invasive continuous blood pressure and ECGs of 37 DCM patients and 167 controls measured under standard resting conditions were analysed. The results show highly significant differences between DCM patients and controls in heart rate and blood pressure variability as well as in baroreceptor sensitivity parameters. Applying a combined heart rate-blood pressure trigger, ventricular premature beats were detected in 24.3% (9) of the DCM patients and 11.3% (19) of the controls. This fact demonstrates the limited applicability of short-term HRT analyses. However, the HRT parameters showed significant differences in this subgroup with ventricular premature beats (turbulence onset: DCM: 1.80±2.72, controls: - 4.34±3.10, p<0.001; turbulence slope: DCM: 6.75±5.50, controls: 21.30±17.72, p=0.021). Considering all (including HRT) parameters in the subgroup with ventricular beats, a discrimination rate between DCM patients and controls of 88.0% was obtained (max. 6 parameters). The corresponding value obtained for the total group was 86.3% (without HRT parameters). Comparable classification rates and high correlations between heart rate turbulence and variability and baroreflex parameters point to a more universal applicability of the latter methods.

## Introduction

Analysis of blood pressure (BPV) and heart rate variability (HRV) is gaining importance in the risk ratification of patients after myocardial infarction  [1] and with a reduced left-ventricular ejection function [2,3]. A decreased HRV as well as an increased BPV represent an adverse prognosis [4,5]. Besides the investigation of individual signals, analysis of the coupling between blood pressure and heart rate, i.e. the baroreceptor sensitivity (BRS), yields major additional information [6]. A low BRS of patients suffering from a coronary cardiac disease and a reduced left-ventricular function has an important predictive value [7-9]. The heart rate turbulence (HRT) method represents a significant extension of the variability and BRS analyses [10]. It was demonstrated that the counter-regulation of the NN intervals (beat to beat) directly after ventricular premature beats (VPB) yields further information for the prognosis following a myocardial infarction. The dominating HRT parameters describe the decrease of the NN interval after the premature beat (turbulence onset) as well as the subsequent increase in the NN intervals (turbulence slope). This regulation mechanism is assumed to be related to baroreflex (BR) regulation [11]. HRT analysis carried out preferably with 24-hour ECGs. However, shorter measurement durations are not excluded by the methodology [11-15].

This study was aimed at investigating the suitability of short-term heart rate turbulence analysis in comparison to variability and spontaneous baroreceptor sensitivity analysis for characterising patients with dilated cardiomyopathy (DCM) and healthy controls. A correlation analysis was applied to identify parameters that register similar dynamic phenomena as the HRT and, hence, might possess the same prognostic value irrespective of the occurrence of ventricular premature beats. The discrimination rates of HRV, BPV, and BRS analysis methods to separate both groups shall be determined and compared to those of HRT analysis. The study was based on short term measurements, because non-invasive continuous blood pressure measurements over 24 hours are inappropriate here. Furthermore, it has already been demonstrated repeatedly that short-term analyses may have a high prognostic value [5] [16].

## Methods

### Patients

Within the framework of the study, 37 patients suffering from idiopathic DCM (male: 27; female: 10; average age: 50.6±12.5 years) and a group of 167 controls (male: 109; female: 58; average age: 43.4±14.7 years) were examined. Due to the reduced number of ventricular premature beats expected, the number of healthy controls was increased.

The DCM patients suffered from a significant dilation of the left ventricle with a limited left-ventricular ejection fraction of 29.5±11.2% (NYHA classes II-III). For medication, all 37 patients were given ACE inhibitors, 16 patients were prescribed betareceptor blocker. 29 patients were additionally treated with diuretics.

Continuous blood pressure of all patients and controls was recorded using a noninvasive photoplethysmographic device of the type PORTAPRES (by TNO-BMI, the Netherlands) via a finger cuff (30 minutes, 100 Hz sampling frequency, under resting conditions). As a reference, a standard ecg was recorded synchronously. All examinations were made under comparable ambient conditions (place, time).

The systolic and diastolic blood pressures as well as the beat-to-beat intervals of the heart were extracted using the system-inherent program “Beatfast” (TNO-BMI). Prior to HRV and BPV analysis, the data were pre-processed using a filter to minimise the influence of premature beats and reduce artefacts and noise [17]. For baroreflex and HRT analysis, unfiltered data were applied. Statistical analysis was performed by the non-parametric Mann-Whitney-U test and subsequent Bonferroni correction. By 6 stepwise discriminant analysis, the individual parameters were checked for suitability for classifying DCM patients and controls. Furthermore, a correlation analysis (Pearson) was made to determine linear dependencies among the HRV, BPV, BRS, and HRT parameters.

### Determination of Blood Pressure and Heart Rate Variability

To analyse the dynamics of the registered time series of systolic (SBP) and diastolic blood pressure (DBP) as well as of beat-to-beat intervals (NN intervals), a number of already established and new methods were applied [17]. HRV, systolic (SBPV), and diastolic blood pressure variability (DBPV) were analysed by time and frequency domain methods as well as by methods of non-linear dynamics. In the time domain, the following parameters were calculated from the filtered data series (NN intervals) [17]: 'meanNN', 'sdNN', 'pNN50' (only HRV) and 'rmssd' [18]. 'meanNN' denotes the mean value of the filtered time series, 'sdNN' the standard deviation, 'pNN50' the relative proportion of NN interval differences larger than 50 milliseconds, and 'rmssd' the root of the squares of successive NN interval differences. In the frequency domain, the frequency bands of 'VLF', 'LF', 'HF', and 'P' were calculated. The 'Very Low' frequency range 'VLF' extends from 0.0033 to 0.04 Hz, the 'Low' frequency range 'LF' from 0.04 to 0.15 Hz, the 'High' frequency range 'HF' from 0.15 to 0.4 Hz, and the total frequency range 'P' from 0 to 0.4 Hz. Analysis also included the following quotients: 'VLF/P', 'HF/P', and 'LF/HF' as well as 'LFn', the normalised 'LF' band [18]. In addition, special non-linear parameters of symbolic dynamics were calculated ('forbword', 'fwshannon', and 'plvar10') [16,19,20]. Symbolic dynamics, a method for the analysis of complex systems, allows to study dynamic aspects of HRV and BPV [8,20].

### Determination of Spontaneous Baroreceptor Sensitivity

Spontaneous BR regulation was carried out using the Dual Sequence Method (DSM) [21]. Compared to the classical sequence method, this method has the advantage of the analysis ranges being extended. Apart from bradycardic, synchronous variations of the NN intervals at increasing blood pressures, also tachycardic as well as delayed reactions of the NN intervals to SBP fluctuations can be analysed. It was demonstrated that these extended ranges yield additional information on BR regulation as well as on the relationships between respiration and BR regulation  [21]. BRS was calculated in the following ranges:

'brady_sync': (increasing SBP causes synchronously increasing NN intervals)

'brady_shift': (increasing SBP causes a delayed increase in NN intervals)

'tachy_sync': (decreasing SBP causes synchronously reduced NN intervals)

'tachy_shift': (decreasing SBP causes delayed reduction of NN intervals)

The number of baroreflex fluctuations (3 consecutively increasing or decreasing values of SBP and NN intervals, >5 ms/mmHg) was determined over 30 minutes. In addition, it was normalised to the total number of heart beats (parameter: ‘norm. number’). Moreover, the average slope (parameter: ‘av. slope’) was calculated by linear regression [22].

Furthermore, other parameters were calculated, by means of which the probability of occurrence of BR fluctuations can be expressed in relation to the SBP fluctuations causing them. This is necessary to analyse the relation between the changed SBP variability and the occurrence of spontaneous BR. The parameters 'P_brady' and 'P_tachy' characterise the frequency of increasing or decreasing SBP triples in relation to the total number of values [21]. In this way, it is possible to estimate whether a changed number of spontaneous SBP fluctuations already causes a changed BR fluctuation number. The relation of how many SBP fluctuations cause a BR is described by the parameter 'P_BR'. This parameter is defined as:

P_BR=(BR_number/SBP_fluctuations) x 100%

### Determination of  Heart Rate Turbulence

To analyse HRT, the classical NN interval trigger was extended for the detection of ventricular premature beats (VPB) [10]. VPB detection by the classical trigger is based on the fact that the premature beat has to be smaller than 80% and the following post-extrasystolic pause has to exceed 120% of the last five normal beats (under the condition: NN intervals >200 ms, NN intervals < 2000 ms). Another pre-requisite is that the 5 pre- and 18 post-extrasystolic heart beats are normal beats (post-extrasystolic: NN intervals after the compensatory pause). When using this trigger, however, arrhythmias, artefacts, and extraction errors of the non-invasive blood pressure measurement device were found just in this range prior to and after the VPB. For this reason, an automatic method was introduced, which facilitates VPB evaluation (see Appendix).

Besides the classical parameters of turbulence onset ('TO') and turbulence slope ('TS') [10], a number of other parameters were calculated. They were used to normalise the HRT parameters with respect to the SBP and DBP values. The parameter ‘TS’ was extended to 'TS_C_' by adaptation to prematureness, the compensatory pause, and the normal values prior to the VPB [15,23].

TS_C_ = (TS x NN_Norm_)/(NN_i+1_-NN_i_) (turbulence slope corrected)

Moreover, new parameters were developed from the parameter ‘TS’, which represent the relation of the SBP and DBP values during the premature beat. These parameters may be presented as follows:

TS_C1_ =NN_i_/SBP_i_ x TS

(adaptation of TS to prematureness and the resulting SBP)

TS_C2_ =NN_i+1_/SBP_i+1_ x TS

(adaptation of TS to the compensatory pause and the resulting SBP)

TS_C3_ =TS/(SBP_i_-DBP_i_)

 (adaptation of TS to SBP and DBP during the premature beat)

 TS_C4_ =TS/(SBP_i+1_-DBP_i+1_)

 (adaptation of the TS to SBP and DBP during the compensatory pause)

## Results

### Analysis of Heart Rate and Blood Pressure Variability

From the data registered for the complete group, the HRV, SBPV, and DBPV were calculated first. A selection of the parameters calculated in these three ranges is presented in Table 1. The table contains those parameters, the uncorrected significance levels of which are below p<0.001 (Table 1). The results of the variability analysis confirm a significant shift of the HRV in all analysis ranges. For the BPV, mainly the frequency range parameters exhibit significant changes.

### Analysis of Spontaneous Baroreceptor Sensitivity

Spontaneous baroreceptor sensitivity was determined by the Dual Sequence Method also for the complete data base. Significant results are listed in Table 2. The number of BR-causing SBP fluctuations ('P_brady' or 'P_tachy') is significantly reduced in DCM patients. Compared to the healthy controls, the reflex is activated at about 50% of the SBP fluctuations only ('P_BR'). This is particularly obvious in case of a longer and stronger counter-regulation (Brady_shift: 'P_BR'). It results in a reduced number of BRS fluctuations in particular for a stronger regulatory activity (brady_shift). The average slope in BR is significantly reduced in DCM patients.

### Analysis of Heart Rate Turbulence

Using the NN interval BP trigger described, suitable VPB were detected in 19 of the 167 controls (11.3%) and 9 of the 37 DCM patients (24.3%). As too many arrythmias (no 18 postextrasystolic normal beats) or very large blood pressure variabilities were observed in some DCM patients in particular, the numbers of analysed VPB of DCM patients and VBP controls differed slightly only (number of VPB DCM: 2.00 ± 1.85; controls: 2.5 ± 2.01; n.s.). The calculated HRT parameters are listed in Table 3.

The ‘TO’ of DCM patients is significantly increased as compared to that of the controls. For the latter, however, a considerable increase in the NN intervals can be noticed after the premature beat. Apart from the parameter 'TS', the corrected parameters 'TS_C_' and 'TS_C1_' are most reduced in DCM patients.

### Correlation Analysis Between HRT and Variability Prameters

Correlation analysis was carried out for the subgroup with VPB consisting of 9 DCM patients and 19 controls to determine linear dependencies between HRT and other analysis methods. The parameter ‘TO’ obviously is negatively correlated with the LF parameters of blood pressure variability and the number of baroreflex fluctuations ( Table 4). In addition, a negative correlation with BR fluctuation in high slope ranges was found.

The HRT parameter ‘TS’ was found to be positively correlated with the HRV and BRS parameters ( Table 5). A correlation analysis that was carried out separately for DCM patients and controls yielded even larger correlations for some parameters.

### Discrimination Function Analysis

Stepwise discriminant analysis was applied to estimate the suitability of the individual analysis methods for the best possible discrimination between DCM patients and controls. For comparison, the analysis was carried out for the complete group and the subgroup with VPB using a maximum of 6 parameters each. By cross-validation, the parameter sets obtained were checked for classification accuracy.

For the discrimination of the complete group (set 1), the following parameters were determined: *Parameter set 1*: 'HFn' (DBPV), 'HF/P' (DBPV), 'VLF/P' (DBPV), 'noNNtime' (HRV), 'brady_shift 6-9_percent' (BRS), 'brady_shift 9-11_percent' (BRS).

The best parameters for discriminating between DCM patients and controls are frequency domain parameters in the DBP, the number of filterings in the HRV when detecting VPB as well as the percentage of BRS fluctuations, at which the heart reacts to stronger blood pressure increases (brady_shift) by 6-9 ms/mmHg and 9-11 ms/mmHg. With this set of 6 parameters, a discrimination rate of 86.3% was reached. 29.7% of the DCM patients and 10.2% of the controls were assigned to the other group, respectively.

For the discrimination of the subgroup with VPB, the following set 2 was determined to be the best possible parameter combination: *Parameter set 2*: 'tachy_shift 5-7_number (BRS), 'brady_shift 6-9_percent (BRS), 'brady_sync 13-15_number (BRS), 'TS_C_' (HRT), 'FWRenyi4' (DBPV), 'renyi4' (DBPV).

These parameters represent the numbers of BRS fluctuations in low (5-7 ms/mmHg) and higher ranges (13-15 ms/mmHg), the 'TS_C_' value normalised to prematureness, pause, and normal NN intervals as well as the time domain parameters of the Renyi entropy in DBP that are measures of the variation range of the DBP. For this subgroup with VPB, a best possible discrimination of 88.0% was calculated. 10% of the DCM patients and 13.3% of the controls were misidentified.

## Discussion

Analysis of variability parameters, baroreceptor sensitivity, and heart rate turbulence is gaining importance in the characterisation of cardiovascular diseases, risk stratification, and the prognosis of the course of the disease [9,15,24,25,26]. The present study was aimed at comparing the recently introduced HRT parameters with the classical variability parameters and baroreceptor sensitivity in terms of applicability, discrimination behaviour, and their correlations for both DCM patients and controls. The major limitation of this study is the short term analysis of the HRT in a time window of 30 minutes. However, the expected inaccuracy of non-invasive continuous blood pressure measurement in long range time windows and the intended analysis of HRT, BPV and BRS couplings explained the introduced study design.

The study confirms the highly significant differences between DCM patients and controls in cardiovascular regulation. The results confirm that an increased heart rate of DCM patients, caused by an enhanced sympathetic activation, induces a reduced HRV. As reported in [2], the LF fluctuations (0.1 Hz) of the NN intervals and SBP and DBP signals observed in controls significantly exceed those of DCM patients. Reduced LF variability also indicates an enhanced sympathetic activation [3] which obviously is directly associated with the vagal suppression found by BRS analysis. Vagal suppression may possibly cause the limited heart rate dynamics of DCM patients. It is quantified by the entropy of the word distribution of 'fwshannon'. The SBP of the controls is higher than in DCM patients. Variability is increased due to the existing LF fluctuations among others.

According to BRS analysis, frequency of occurrence of BR fluctuations and their sensitivity are significantly reduced in DCM patients. Obviously, blood pressure variability that causes the BR is already changed primarily in DCM patients. Whereas the controls exhibit about 19% increasing BP fluctuations (3 consecutive increasing blood pressure values / total number of values), the percentage only amounts to about 14.5% for DCM patients. This suggests that a smaller number of BR activations is to be expected due to the reduced number of SBP fluctuations. Moreover, the BR is less often induced in DCM patients at the given number of SBP fluctuations. This particularly applies to stronger SBP fluctuations (parameter 'brady_shift'), where a BR is induced in DCM patients at 8.7% of the SBP fluctuations only. For the controls, this value amounts to about 15.9%. BR regulation of DCM patients is reduced not only due to the reduced SBP fluctuations, but also because it less often reacts at the given SBP fluctuations. Compared to healthy controls, the response rate is reduced by about 50% for stronger fluctuations. Comparison of the average slopes in BR also indicates a vagal suppression. In DCM patients, the BR reacts more weakly, such that stronger blood pressure fluctuations obviously are not compensated by BR heart rate adaptation. The relatively high average slopes in DCM patients compared to the values given in literature [27] and own analyses [21] are due to the small resolution of the blood pressure measurement device PORTAPRES of f_A_=10 ms and unfiltered data analysis. Fluctuations smaller than 10 ms were not detected.

VPB detection has shown that HRT methods can be applied to a subgroup only, as ventricular premature beats are lacking or occur too frequently. VPB suited for analysis were found in 24.3% of the patients and 11.3% of the controls only. Dependence on VPB occurrence and their usability for short-term analysis represent considerable limitations of the HRT method. For the subgroup with VPB, however, the classical HRT parameters revealed highly significant differences between the groups examined. While the NN intervals after the VPB increased considerably in the patients, they decreased in the controls. Moreover, DCM patients showed a significantly reduced 'TS' [28].

The recently introduced parameter of pulse amplitude potentiation in the SBP after a VPB, 'PEAP' [13,29], did not exhibit any significant changes for our group of patients. This parameter that characterises the hemodynamics during ventricular premature beats did not exhibit any significant differences. The new trigger applied for the detection of VPB may influence this parameter, as a reduced number of VPB only is included in the analysis. The assumption [13,29] of the 'PEAP' influencing the baroreflex regulation cannot be supported by the present study due to the very small correlation of r=-0.11 (r_max_=0.44; r_min_=-0.48) with the BRS parameters.

Stepwise discriminant analysis was applied to identify the 6 parameters that are best suited for discriminating between the subgroup with VPB and the entire group without HRT parameters. In parameter set 1 for the discrimination of the entire group, DBPV is the dominating parameter. The HRV parameter 'noNNtime' represents the number of premature beats filtered out prior to variability analysis. This suggests a high significance of the number and expression of VPB, even if morphology is not studied explicitly. Both BRS parameters characterise larger NN fluctuations that still have a counter-regulating effect after three values following the SPB fluctuations. The lower (6-9 ms/mmHg) and higher slope ranges (9-11 ms/mmHg) are analysed. They behave reciprocally. Discrimination of the subgroup with VPB (*parameter set 2*) does not only include the BRS parameters over low and very high slope ranges, but also those of the DBPV. Moreover, the HRT parameter 'TSC' normalised to prematureness and pause is included. During automatic selection of the parameters by discriminant analysis, it became obvious that the largest share in the information is supplied by the DBPV and BRS parameters, as these are available in the parameter set of the subgroup with VPB as well as for the complete group.

Correlation analysis was made to determine the relationships between HRT and the prognostically significant variability and BRS parameters [5] that may be obtained by a VPB-independent analysis. It must be taken into account, however, that arrhythmias (e.g. VPB) were filtered out prior to HRV and BPV analysis, whereas unfiltered data were used for HRT and BRS calculation. The high negative correlations between the HRT parameter 'TO' and the LF blood pressure parameters suggest a relationship with sympathetic activation [30]. Obviously, the small expression of LF fluctuations is associated with an increase in 'TO'. The high slope ranges (between 12 and 18 ms/mmHg) of BRS also correlate with the 'TO'. A high onset (<0) is related with a reduced BRS. Obviously, the heart of the DCM patients cannot compensate stronger blood pressure fluctuations by a strong and rapid heart rate adaptation.

In addition, high correlations between the HRT parameter 'TS' and several HRV parameters were calculated. Anyhow, the large slopes in the baroreflex correlate with 'TS' [14,15,31]. The high correlation between 'TS' and the 'LF' HRV parameter suggests a relationship with this parameter established for risk stratification [30].

The study is limited by the small number of VPB recorded over the measurement period (30 minutes) and the resulting restriction of the examined group. Although the HRT analysis method was not applied exclusively to 24-hour ECG measurements [13,29], more suited VPB are to be expected over longer measurement durations. In addition, the study results are negatively affected by the fact that due to the small number of male and female patients and controls in the subgroups with VPB, no distinction was made, although there are sex-specific differences in cardiovascular regulation [21]. Moreover, the results, according to which the BRS is reduced in DCM patients with a strongly limited ejection fraction (EF ~ 29%), are not totally unexpected [27,32]. However, our study was primarily aimed at estimating the quality of the different methods in the characterisation of this disease.

Furthermore, it was not distinguished between the treatments of the patients. It may be assumed that vagal suppression in DCM patients is not significantly influenced by the beta-blocker therapy. Possibly, the reduced vagal or increased sympathetic activation would be further enhanced without beta-blocker therapy [33].

To sum up, the HRT shows highly significant differences in the extended characterisation of DCM patients and controls, for which a number of VPB was recorded. Over a period of 30 minutes, however, this number was rather limited such that HRT analysis could be applied to 14% of the entire group only. Compared to other parameters, HRT analysis hardly succeeded in improving the discrimination rate. The high correlations between the HRT and the BPV, HRV, and BRS parameters suggest that similar dynamic phenomena of cardiovascular regulation are recorded, which, however, are independent of the occurrence of VPB. In view of the high correlations of the HRV, BPV, and BRS parameters with HRT and the good discrimination rate for the entire group examined, a future clinical study should be aimed at verifying the prognostic value of the variability parameters and baroreceptor sensitivity.

From the results obtained, it may be concluded that HRT analysis can be applied in short-term investigations (e.g. screenings) with certain restrictions only. Consequently, HRT should preferably be applied to long-term examinations with numerous VPB expected, while variability and baroreflex analyses may be applied more universally.

## Figures and Tables

**Figure 1:**
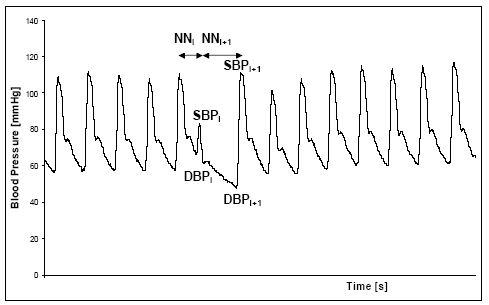
Schematic representation of the characteristics of the NN intervals (NN), systolic (SBP), and diastolic blood pressure (DBP) during the VPB using the blood pressure of a DCM patient as an example

**Table 1:**
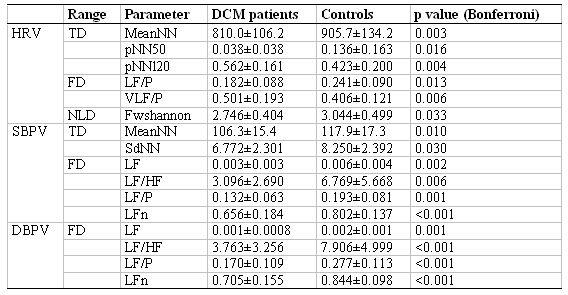
Significantly different parameters of heart rate (HRV) and systolic (SBPV) and diastolic blood pressure variability (DBPV) in patients with DCM and controls (TD: time domain parameters, FD: frequency domain parameters, NLD: non-linear dynamics parameters)

**Table 2:**
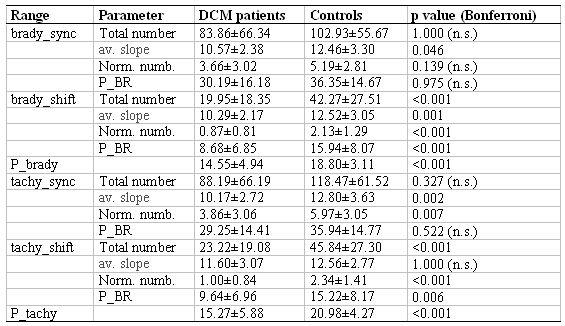
Baroreflex behaviour of DCM patients and controls, as obtained by the Dual Sequence Method (the DSM analysis ranges are explained in the section “Methods”)

**Table 3:**
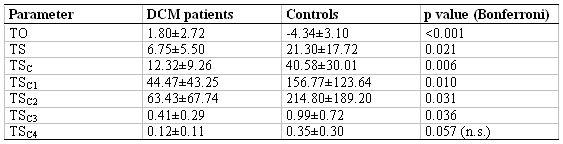
Differences of the heart rate turbulence and extended parameters for a subgroup of 9 DCM patients and 19 controls

**Table 4:**
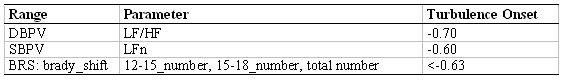
Correlation analysis of the HRT parameter 'Turbulence Onset' with the LF parameters of blood pressure variability and baroreceptor sensitivity ('12-15_number' represents the number of BR fluctuations between 12 and 15 ms/mmHg or 15 and 18 ms/mmHg, or the total number of baroreflex fluctuations, p<0.01)

**Table 5:**
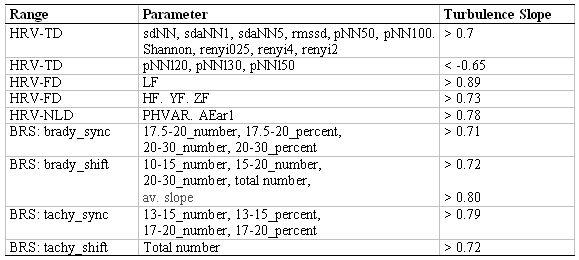
Correlation analysis of the HRT parameter 'Turbulence Slope' with the variability parameters and baroreceptor sensitivity (the BRS parameter '13-15_percent' represents the percentage of BR fluctuations lying between 13 and 15 ms/mmHg relative to the BR fluctuation number, p<0.01)

## Appendix

To prevent misdetections of VPB for HRT analysis, a combined HRV-BPV trigger was developed that particularly accounts for the standard behaviour of the 5 pre- and 18 post-extrasystolic SBP values as well as their NN interval values. As shown in Figure 1, the time 'i' represents the time of the shortened extrasystolic beat. All further conditions are referred to this time. The normalised NN interval 'NN_Norm_' represents the arithmetic mean of the 5 pre-extrasystolic normal beats (NN_Norm_=mean(NN_i-5_,…,NN_i-1_)).

The main conditions correspond to the classical arrhythmia trigger for VPB [10] and may be expressed as follows:

'NN_i_': NN interval < 80% x NN_Norm_;

This condition corresponds to the prematureness of the ventricular beat, while the following definition represents the compensatory pause:

'NN_i+1_': NN interval > 120% x NN_Norm_;

To meet the extended conditions to ensure the 5 pre- and 18 post-extrasystolic normal beats, the following definitions are required:

SD _NN PRE_ := SD (NN_i-5_, …, NN_i-1_);

Standard deviation of the NN intervals prior to the VPB

SD _NN POST1_ := SD (NN_i+2_, …, NN_i+6_);

Standard deviation of the NN intervals of 5 beats after the compensatory pause

SD _NN EXT_ := SD (NN_i-5_, …, NN_i+6_);

Standard deviation of the NN intervals of 5 pre- and 5 post-extrasystolic beats, taking into account the VPB

SD _SBP EXT_ := SD (SBP_i-5_, …, SBP_i+6_);

Standard deviation of the SBP of 5 pre- and 5 post-extrasystolic beats taking into account  the VPB

SD _NN POST2_ := SD (NN_i+7_, …, NN_i+19_);

Standard deviation of the NN intervals of the 5th through 18th post-extrasystolic beat

SD _SBP POST2_ := SD (SBP_i+7_, …, SBP_i+19_);

Standard deviation of the SBP of the 5th through 18th post-extrasystolic beat

For the detection of the VPB, the following conditions have to be fulfilled:

Condition 1: (1.5 x SD_NN PRE_ < SD_NN EXT_)

Condition 2: (1.5 x SD_NN POST1_ < SD_NN EXT_)

Condition 3: (1.5 x SD_NN POST2_ < SD_NN EXT_)

Condition 4: (1.5 x SD_SBP POST2_ < SD _SBP EXT_)

Condition 5: (SBP_i_ < 0.9 x SBP_i-1_)

Condition 6: (SBP_i-1_ < 1.2 x SBP_i-2_)

Condition 7: (SBP_i+1_ > SBP_i_)

Conditions 1-3 ensure that only normal beats occur prior to and after the VPB without suppressing potential fluctuations of the NN intervals. Conditions 4-7 describe the blood pressure behaviour prior to, during, and after the ventricular premature beat. Here, it is taken into consideration that the premature beat builds up a smaller SBP only.
